# A Novel B-Cell Epitope Identified within *Mycobacterium tuberculosis* CFP10/ESAT-6 Protein

**DOI:** 10.1371/journal.pone.0052848

**Published:** 2013-01-07

**Authors:** Hua Yang, Haizhen Chen, Zhonghua Liu, Hui Ma, Lianhua Qin, Ruiliang Jin, Ruijuan Zheng, Yonghong Feng, Zhenling Cui, Jie Wang, Jinming Liu, Zhongyi Hu

**Affiliations:** 1 Shanghai Key Laboratory of Tuberculosis, Shanghai Pulmonary Hospital, Tongji University School of Medicine, Shanghai, China; 2 Department of Respiratory Medicine, Shanghai Pulmonary Hospital, Tongji University School of Medicine, Shanghai, China; 3 Clinical Laboratory Diagnostics, Shanxi Medical University, Taiyuan, China; National Institute of Infectious Diseases, Japan

## Abstract

**Background:**

The 10-kDa culture filtrate protein (CFP10) and 6-kDa early-secreted target antigen (ESAT-6) play important roles in mycobacterial virulence and pathogenesis through a 1∶1 complex formation (CFP10/ESAT-6 protein, CE protein), which have been used in discriminating TB patients from BCG-vaccinated individuals. The B-cell epitopes of CFP10 and ESAT-6 separately have been analyzed before, however, the epitopes of the CE protein are unclear and the precise epitope in the positions 40 to 62 of ESAT-6 is still unknown.

**Methods:**

In the present study, we searched for the B-cell epitopes of CE protein by using phage-display library biopanning with the anti-CE polyclonal antibodies. The epitopes were identified by sequence alignment, binding affinity and specificity detection, generation of polyclonal mouse sera and detection of TB patient sera.

**Results:**

One linear B-cell epitope (KWDAT) consistent with the 162^nd^–166^th^ sequence of CE and the 57^th^–61^st^ sequence of ESAT-6 protein was selected and identified. Significantly higher titers of E5 peptide-binding antibodies were found in the sera of TB patients compared with those of healthy individuals.

**Conclusion:**

There was a B-cell epitope for CE and ESAT-6 protein in the position 40 to 62 of ESAT-6. E5 peptide may be useful in the serodiagnosis of tuberculosis, which need to be further confirmed by more sera samples.

## Introduction

Tuberculosis (TB) remains one of the major global health problems, particularly in most developing countries. Estimates are that one-third of the world's population is currently infected with *Mycobacterium tuberculosis* (MTB). In 2010, there were 8.8 million incident cases of TB and 1.45 million deaths from TB [1]. Early detection of TB is especially important in order to provide early treatment and curtail the spread of infection. However, in developing countries, the diagnosis of TB still largely depends on the isolation of the slow-growing MTB [2]. Compared with the conventional bacterial culturing which takes up to 6–8 weeks, antibody detection tests (predominantly serological tests) are simple, rapid and require only a single visit to the clinic [3]. Serological tests that are currently commercially available for TB diagnosis continue to produce inconsistent and imprecise estimates of sensitivity and specificity, however, and a recent meta-analysis of these tests developed very poor quality of evidence to support diagnosis [4]. WHO warned against the utility of current serological tests in the immunodiagnosis of TB and strongly recommended that they must not be used for the diagnosis of pulmonary and extra-pulmonary TB [5]–[6]. Further research to identify new/alternative point-of-care tests for TB diagnosis and/or serological tests with improved accuracy is strongly encouraged by WHO [5].

The 10-kDa culture filtrate protein (CFP10) and 6-kDa early-secreted target antigen (ESAT-6) are two low molecular weight secretory proteins encoded by the Rv3874 and Rv3875 gene, respectively. These genes are located in the region of difference-1 (RD-1) of the MTB genome, but are absent in all *Mycobacterium bovis* bacillus Calmette-Guerin (BCG) vaccine strains [7]. It is well established that CFP10 and ESAT-6 are important in mycobacterial virulence and pathogenesis, and play a key role in pathogen to host cell signaling [8]. Since CFP10 and ESAT-6 are not present in BCG and many atypical mycobacteria, they have been useful in discriminating TB patients from BCG-vaccinated individuals and individuals exposed to non-tuberculous mycobacteria (NTM) [9], [10]. Kunnath-Velayudhan *et al.* defined the MTB immunoproteome using high-throughput detection of antibodies in human serum against the entire MTB proteome. This is rich in membrane associated and extracellular proteins and 13 of these proteins, including CFP10, were found to have a significant association with active TB [11]. Research of Hoff ST also confirmed the presence of antibodies against ESAT-6 and CFP10 in patients with TB, and demonstrated that significant antibody responses are not restricted to active TB disease but can also reflect latent infection, particularly in areas with high levels of exposure to MTB [12]. Using overlapping peptides and enzyme-linked immunosorbent assay (ELISA), Harboe *et al.* [13] obtained a precise mapping of the B-cell epitope at positions 3 to 15 on the ESAT-6 molecule and also confirmed that antibodies formed by prolonged immunization with a 23-mer peptide (positions 40 to 62) could react with native ESAT-6, although the precise epitope in this region is still unknown. Spencer *et al.* comparatively analyzed the B- and T-cell epitopes of CFP10 of *Mycobacterium leprae* and MTB, but no clear mapping of the B-cell epitopes of CFP10 of MTB was obtained [14]. Importantly, it has been shown a tight 1∶1 complex and stable tertiary structure are formed between CFP10 and ESAT-6, and this complex represents the functional forms of CFP10 and ESAT-6 [15], [16]. Analyses of immune responses against the complex, known as the CE protein, have been limited.

New strategies have been tested in recent years to investigate and characterize regions with IgG-binding capacity. For example, using antigen microarray, Nahtman *et al.* [17] screened synthetic peptides spanning multiple MTB proteins, including CFP10 and ESAT-6, for serum reactivity. 10 of the 363 peptides assessed had significantly higher responses in the five TB-positive patients analyzed than in control individuals. These included the Q54OD8 peptides (position 53 to 66) of ESAT-6, and POA566 peptides (position 22 to 34) of CFP10, which may represent the B-cell epitope of the two proteins, although this needs to be confirmed.

B-cell epitopes can also now be directly identified using X-ray crystallography of antibody-antigen (Ab-Ag) complexes, but these experiments and methods are not always cost effective, consume time and are not always successful [18], [19]. Alternatively, peptides selected from random libraries displayed on filamentous phage surface proteins pIII or pVIII [20] can be employed for mimotope selection by means of antibody binding and then used for determining the location of immunologically active sites (epitopes) in proteins, carbohydrates, lipids, or as individual epitope-mimicking antigens and immunogens [21], [22]. Mimotopes of several antigens of MTB have been selected successfully before, such as MPT64, lipoarabinomanan (LAM), Hsp16.3, neutral polysaccharides (NPS) and nonprotein antigens. [23]–[27]

In the present report we describe the identification of functional epitopes of CFP10-ESAT-6 (CE) protein by phage display. Experiments with antibodies raised against the mimotope peptides suggested that the identified protein sites may be implicated in the humoral immune response against the CE protein. Sera of TB patients contained significant levels of antibodies that recognized both the peptide and CE protein. Finally, the specificity and sensitivity of a peptide-based ELISA test were found to be similar to those of the CE protein-based ELISA test, indicating that the peptide could be a good substitute for CE for the serodiagnosis of TB.

## Materials and Methods

### Ethics Statement

This study was granted by the Tongji University Ethics Committee (Permit Number: K08-018). All participants provided written informed consents before participation in this study. Animal procedures were approved by the Animal Experimentation Ethics Committee of Shanghai Pulmonary Hospital (Approval ID: SYXK2009-0083).

### Human sera

Venous blood samples were collected from individuals attending the Shanghai Pulmonary Hospital, Shanghai, China. Each TB patient (n = 44) was diagnosed with active pulmonary TB by sputum culture, performed according to the Chinese Laboratory Science Procedure of Diagnostic Bacteriology in Tuberculosis [28]. Blood was collected before the initiation of treatment. No patients presented with systemic or extra-pulmonary TB as assessed by X-ray test. All patients had a history of BCG vaccination and were PPD positive (indurations of >10 mm). The mean age was 31.6 years (range 18 to 71), and the male/female ratio was 4∶1. Blood samples were also collected from 48 healthy individuals who had been vaccinated with BCG during childhood and all were PPD-positive. All study recruits were negative for human immunodeficiency, hepatitis B and hepatitis C viruses. Sera were prepared by centrifugation and stored in aliquots at −70°C until analysis.

To further evaluate the epitope response in the context of latent tuberculosis infection (LTBI), 55 blood samples were collected from individuals recruited based upon their response in the T-SPOT TB test (Oxford Immunotec, Inc.). TB patients (n = 19; fully diagnosed as previously described), LTBIs (n = 18; healthy individuals with positive T-SPOT TB test results) and healthy controls (n = 18; negative by T-SPOT TB test), were kindly presented by Dr. Wei Sha from Shanghai Pulmonary Hospital and tested.

### CE protein and anti-CE polyclonal antibodies(pAbs)

Recombinant fusion protein CE was expressed and purified as a His-tagged protein, as described previously [29]. The pAbs against CE were generated similar to the production of polyclonal anti-MPT64 rabbit antibodies [27]. The titre of the anti-CE antiserum was examined by ELISA and the result was 1∶16000.The anti-CE pAbs were purified by CE-sepharose column chromatography, detected by SDS-PAGE and quantified by Bradford method.

### Phage display selection, DNA sequencing and analysis

Phage display library Ph.D.-7 was purchased from New England Biolabs (Beverly, MA, U.S.A.). The library was screened with anti-CE polyclonal rabbit antibodies according to the manufacturer recommendations (http://www.neb.com/nebecomm/products/productE8100.asp). In brief, purified anti-CE pAbs diluted with 0.1 M NaHCO3, pH 8.6 were coated on the 96-well ELISA plate in 100 µg/ml overnight. After blocking with 0.1 M NaHCO3 (pH 8.6), 5 mg/ml BSA and 0.02% NaN3, the library (2×10^11^ phage for a library with 2×10^9^ clones) was diluted 100-fold with 100 µl of TBST (TBS+0.1% [v/v] Tween-20), added onto coated plate and rocked gently for 60 minutes at room temperature. Unbound phage was discarded and the plate was washed 10 times with TBST. Bound phage was eluted with 100 µl solution of the free anti-CE antibodies (100 µg/ml in TBS) to compete the bound phage away from the immobilized antibodies on the plate. After rocking the elution mixture gently for 60 minutes at room temperature, the eluate was pipetted into a microcentrifuge tube, a small amount (1 µl) of which was titrated and the rest of which was amplified. After determining the phage titer of the amplified eluate and calculating an input volume corresponding to the titer of 2×10^11^, a second round of panning was conducted in a similar manner, using the calculated amount of the first round amplified eluate as the input phage, and raising the Tween-20 concentration in the wash steps to 0.5% (v/v). After the panning was repeated for a total of four rounds, single-stranded DNA of individual isolated phage clones was purified as described by the kit manual and then sequenced with -96gIII primer performed by the Sangon Biotech Co. Ltd. (Shanghai, China). The sequences of the phage displayed peptides were aligned with sequences of CE for homology search using DNASTAR software. Finally, to determine the sites mimicked by the phage displayed peptide sequences on the tertiary structure level, a tri-dimensional model of the CE molecule was generated with PyMOL software.

### Binding affinity and specificity assays

To evaluate the binding affinity and specificity of the selected phages to the purified anti-CE pAbs, individual phage clones with different sequences were amplified according to the manufacturer's instructions. Phage ELISA and competitive ELISA were performed as previously described [27]. Briefly, a 96-well ELISA plate was coated with 100 µg/ml purified anti-CE pAbs overnight at 4°C. Both a coated plate and another without coating (to detect non-specific binding of BSA to the selected phage-displayed peptides), were incubated with blocking buffer. Phage particles were then added to the wells at 10^10^ p.f.u. ml^−1^ and incubated for 1–2 h at room temperature. Subsequently, capture of the phages by antibodies was detected with anti-M13 mAb conjugated to horseradish peroxidase (HRP) (GE Healthcare) using tetramethylbenzidine (TMB) solution as a substrate. The absorbance was measured at 460 and 620 nM using an ELISA plate reader (MK3, Thermo). All samples were tested in triplicate, with the first eluted phage library serving as a negative control and TBST as a blank. Phage clones with more than 3 times higher affinity to anti-CE pAbs than the negative control were defined as positive.

The purified anti-CE pAbs and CE protein were used in a competitive inhibition ELISA to test the specificity of selected phage-peptide clones. In brief, ELISA plates were coated with 100 µg/ml anti-CE pAbs and blocked. 50 µl CE protein at different concentrations (0 g/L, 0.2×10^−3^ g/L, 0.5×10^−3^ g/L,1×10^−3^ g/L, and 5×10^−3^ g/L) and 50 µl phage particles at 10^10^ p.f.u. ml^−1^ were mixed and added to the wells. The ELISA procedure was performed as described above.

### Peptide synthesis

Synthetic peptides carrying amino acid sequence of mimotope (KWDATYTGGGS, designated E5) were prepared by Shanghai Bootech BioScience & Technology Co. Ltd. (Shanghai, China). Biotin-labeled peptides (Biotin-SGGKWDATYTGGGS, designated E5BIO; Biotin-SGGVRWDATTGGGS, designated E6BIO; Biotin-SGGRWDATREGGGS, designated E18BIO) were synthesized for competitive inhibition ELISA of peptides. The structurally flexible linker (SGG or GGGS) was added to the phage displayed peptide sequence at the N-terminal or C-terminal ends to obtain the effective conformations of peptides. For immunization, E5 peptide was also synthesized with the Cysteine substituted Serine linker (GGGC) and conjugated to keyhole limpet hemocyanin (KWDATYTGGGC-KLH, designated E5-KLH).

### Competitive inhibition ELISA of peptides

To test the specificity of each peptide identified on the level of phage displaying, synthetic biotin-labeled peptides and CE, ESAT-6 and CFP10 protein were each used to competitively bind to the anti-CE pAbs. Briefly, 10 µg/ml anti-CE pAbs diluted in coating buffer (0.1 M NaHCO3, pH 9.6) were coated on a 96-well plate overnight at 4°C. After blocking with 1.5% nonfat milk powder in PBS, 50 µl CE, ESAT-6 or CFP10 protein at different concentrations (0 g/L, 0.2×10^−3^ g/L, 0.5×10^−3^ g/L,1×10^−3^ g/L, and 5×10^−3^ g/L) and 50 µl biotin-labeled peptides (E5BIO, E6BIO, E18BIO) at 5 µg/ml were mixed, added to wells and incubated at 37°C for 1 hour. HRP-labeled streptavidin (Sigma) was diluted 1∶2000 and 100 µl per well added for 1 h at 37°C. After developing, the absorbance of each well was measured with an ELISA reader.

### Immunization protocols

Female 6–8 weeks old BALB/c mice were immunized with KLH-conjugated peptide. KLH-conjugated peptide (250 µg in 1.0 ml of PBS) was emulsified with an equal volume of Incomplete Freund Adjuvant (IFA) and administered to 5 mice (50 µg per mouse) by subcutaneously injection two times with a 14-day interval. Control groups were immunized with unconjugated KLH in IFA. For detection of serum-specific anti-E5 peptide antibodies, blood was collected from the tail vein. Then 14 days after the last booster, blood was collected from anaesthetized mice by heart puncture. Sera were prepared and stored in aliquots at −70°C until analysis.

### Indirect ELISA

E5 or CE-binding IgG antibodies were detected by indirect ELISA, as described previously [22]. Briefly, 96-well ELISA plates were coated with 100 µl per well of coating solutions containing either 10^10^ phages ml^−1^, 4 µg peptide ml^−1^ or 1 µg CE protein ml^−1^ overnight at 4°C. After washing and blocking with 1.5% nonfat milk powder in PBS for 1 h at 37°C, sera or anti-E5 IgG (mice sera prior to and after each immunization) diluted 1∶100 in PBST (PBS+0.1% Tween-20 ) was added and incubated for 1 h at 37°C. After washing 3 times, HRP-labeled goat anti-human Ig or anti-mouse Ig (Sigma) was added to each well for 1 h at 37°C, followed by development by the addition of TMB substrate. The absorbance of each well was then measured with an ELISA reader.

### Statistical analysis

Data were analyzed by unpaired T test or Mann-Whitney test, two-tailed. One-way analysis of variance (ANOVA) and Tukey's multiple comparison tests were used for data more than two groups. P values<0.05 were considered to be significant. The receiver operating characteristic (ROC) curve was used to evaluate the performance of the ELISA tests with two categories (TB patients and healthy controls).

## Results

### Phage display screening and peptide analysis

**Figure 1:**
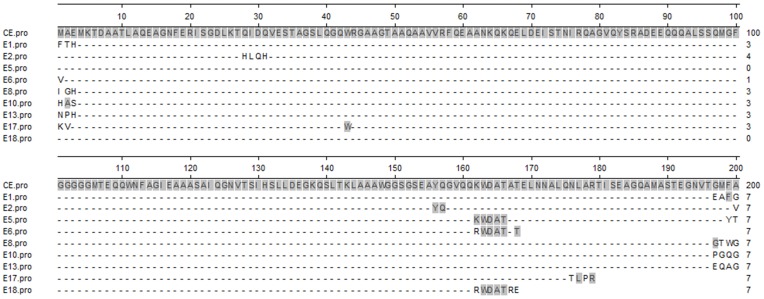
Alignment of selected phage clones to the CE amino acid sequence. Individual phage clone peptide sequence was aligned to the CE sequence (1–200 amino acids) using an alignment software (DNASTAR). Consensus amino acid sequences between individual phage clone peptide sequences and CE sequence are highlighted with solid light gray shade.

**Figure 2:**
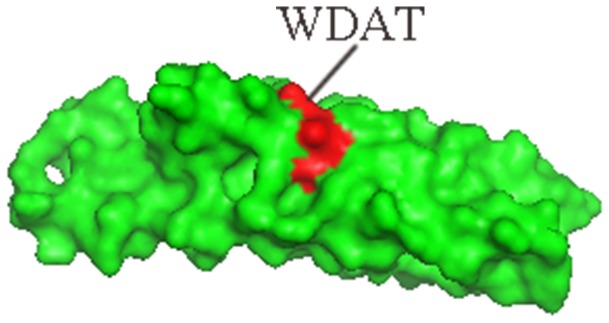
Structure modeling figure of CE protein and WDAT amino acids. The structure modeling figure of CE protein was analyzed with PyMOL software (www.pymol.org), and the W_163_D_164_A_165_T_166_ amino acids (red part) was located on the surface of the space structure of CE.

To find the epitopes or mimotopes of the CE protein, a random 7 peptide phage library was used for the binding analysis of IgG obtained from rabbits immunized with the CE protein. The number of eluted phages bound to IgG gradually increased with successive biopanning rounds (from 1×10^3^ of first round to 2×10^4^ of fourth round). After the fourth round of panning, the peptide-coding regions of 18 randomly selected phage clones were sequenced (Table 1). Nine unique sequences were obtained, and 6 of the 18 phage clones had the same sequence FTHEAFG with highest frequency. Four clones had unique sequences, while clones E2 and E4, E5 and E7, E6 and E9, E8 and E14 separately contained the same sequences, respectively. After alignment of the 9 sequences of these peptides against the amino acid sequence of CE protein, one consensus amino acid sequence WDAT contained by clones E5,E6,E7,E9 and E18 was obtained, which was totally consistent with the 163^rd^–166^th^ sequence of CE (Figure 1) and the 58^th^–61^st^ sequence of ESAT-6 protein. For structure analysis, the modeling figure of CE protein was generated with PyMOL software (www.pymol.org), the amino acid sequence WDAT was located on the surface of the space structure of CE (Figure 2).

**Table 1:** Selected phage clones sequences from anti-CE pAbs.

Clone sequence identifier	Nucleoside acid sequence	Amino acid sequence	Frequency
E1,E3,E11,E12,E15,E16	TTTACTCATGAGGCTTTTGGT	FTHEAFG	6/18
E2,E4	CATTTGCAGCATTATCAGGTT	HLQHYQV	2/18
E5,E7	AAGTGGGATGCTACTTATACT	K**WDAT**YT*	2/18
E6,E9	GTTCGTTGGGATGCGACGACG	VR**WDAT**T*	2/18
E8,E14	ATTGGTCATGGGACTTGGGGT	IGHGTWG	2/18
E10	CATGCGAGTCCGGGGCAGGGT	HASPGQG	1/18
E13	AATCCTCATGAGCAGGCTGGT	NPHEQAG	1/18
E17	AAGGTTTGGACGCTGCCTAGG	KVWTLPR	1/18
E18	CGTTGGGATGCTACGAGGGAG	R**WDAT**RE*	1/18

* Consensus amino acid sequences between distinct phage sequences are highlighted in bold.

### Binding affinity and specificity of the selected phage clones

**Figure 3:**
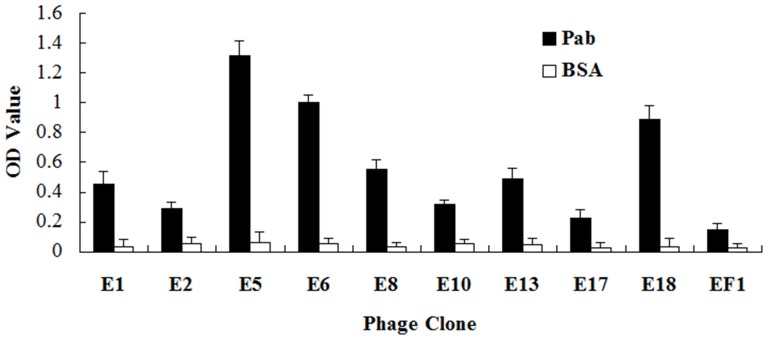
ELISA immunoreactivity of selected phage clones against anti-CE polyclonal antibodies (pAbs) or BSA (control). Nine phage clones with different peptide sequences were used for the phage ELISA analysis. Phage clones were added to the plates coated with either **pAbs** or BSA. Capture of the phages by antibodies was detected with anti-M13 mAb conjugated to horseradish peroxidase using tetramethylbenzidine as a substrate. The reaction was read at 460 and 620 nm. The data represent the mean and standard deviation of triplicate wells. The first eluted phage library (EF1) was used as negative control. PBST (B) was used as blank control. Phage clones E5, E6 and E18 showed more than 4 times higher affinity to anti-CE pAbs, and E1, E8 and E13 also showed more than 3 times higher affinity, all of which should be positive clones.

**Figure 4:**
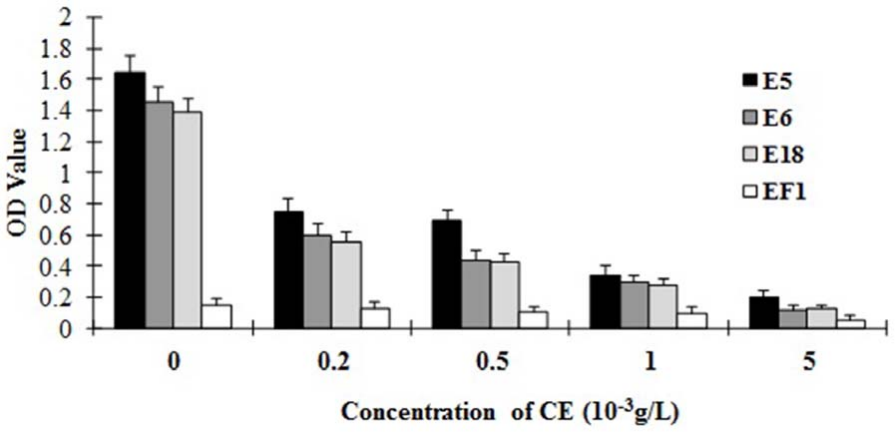
CE inhibitions of E5, E6 and E18 phage clones binding to anti-CE pAbs. Equal volume individual phage clones and CE protein with different concentrations (0 g/L, 0.2×10^−3^ g/L, 0.5×10^−3^ g/L,1×10^−3^ g/L, and 5×10^−3^ g/L) were mixed and added to the plates coated with anti-CE pAbs. Anti-M13 mAb conjugated to horseradish peroxidase was used to detect the phages. ELISA was performed continuingly as described before. The first eluted phage library (EF1) was used as negative control. The CE protein could inhibit the interactions of the phage clones E5, E6 and E18 to anti-CE pAbs and the inhibition was concentration dependent.

**Figure 5:**
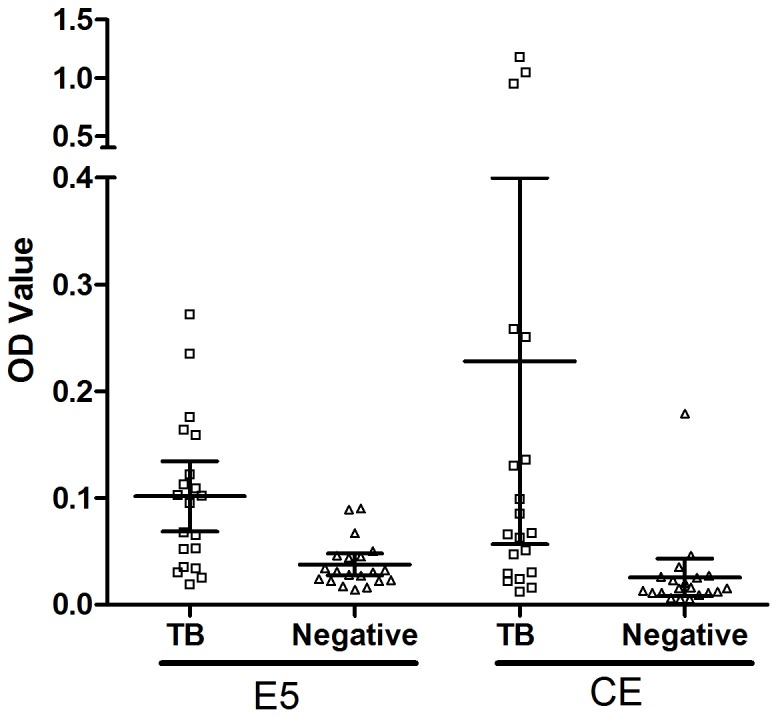
Scatter dot plot representation of the distribution of IgG levels to the E5 phage clone and CE protein in TB patients (□) and negative control (▵). (Error bars: 95% confidence interval for mean) The plates were coated with 10^10^ phages ml^−1^ or 1 µg CE protein ml^−1^ per well, as described in Methods. Sera from TB patients (n = 20) and healthy individuals (n = 20) were tested. The results are shown as the mean OD value of each triplicate sample read 20 min after the addition of substrate. Significance was determined against the negative controls group of E5 phage clone and CE protein (t = 3.877, P = 0.0004; t = 2.460 P = 0.0186).

To confirm the binding affinity of selected phage clones, nine clones displaying different peptide sequences were amplified and tested by phage ELISA. The most efficient clone was E5, although clones E6 and E18 also had more than 4 times greater affinity to the anti-CE pAbs than the first eluted phage library, which was used as negative control. Phage clones E1, E8 and E13 were all positive and showed more than 3 times higher affinity (Figure 3). The binding specificities of the phage clones with high affinity were then tested by competitive ELISA. The results indicated that the CE protein could inhibit the interaction of the phage clones E5, E6 and E18 with the anti-CE pAbs and the inhibition was concentration dependent (Figure 4). To evaluate the binding activities of phage displayed peptides to human sera containing anti-CE antibodies, E5 phage clone and CE protein were used as antigens and tested by ELISA. Randomly selected sera from TB patients (n = 20) and healthy controls (n = 20) were analyzed. Similar to results obtained with the CE protein, a stronger reactivity was observed among TB patients than healthy controls for the E5 phage (t = 3.877, P = 0.0004; t = 2.460 P = 0.0186) (Figure 5).

### Competitive inhibition ELISA of the peptides

**Figure 6:**
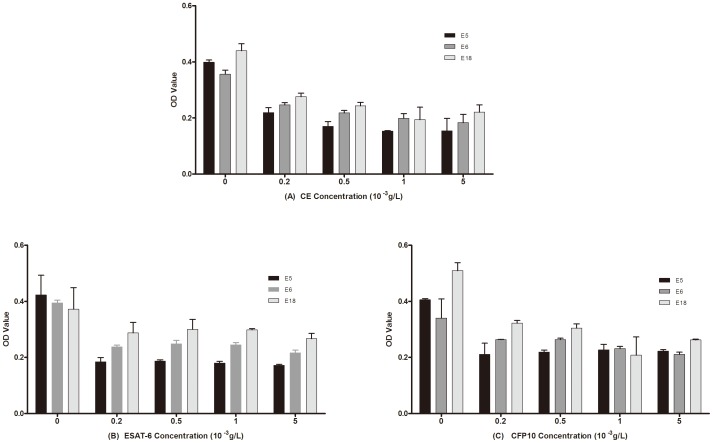
Competitive inhibitions of E5BIO, E6BIO and E18BIO peptides binding to anti-CE pAbs with CE(A), ESAT-6(B) and CFP10(C) proteins, respectively. Equal volume individual biotin-labeled peptides and CE, EAST-6 or CFP10 protein with different concentrations (0 g/L, 0.2×10^−3^ g/L, 0.5×10^−3^ g/L,1×10^−3^ g/L, and 5×10^−3^ g/L) were mixed and added to the plates coated with anti-CE pAbs. Streptavidin conjugated to horseradish peroxidase was used to detect the peptides. ELISA was performed continuingly as described before. Absorbance values of the 3 peptides binding to anti-CE pAbs were all decreased with the adding of any one of the 3 proteins.

Based on the results obtained by screening and identification of selected phage clones, peptides incorporating the amino acids of identified mimotopes (E5, E6, and E18) were synthesized and labeled with biotin. These peptides were then used to test if CE, ESAT-6 or CFP10 protein could competitively inhibit the binding of the mimotope peptides to anti-CE pAbs. Absorbance values of the 3 peptides binding to anti-CE pAbs were all decreased with the adding of any one of the 3 proteins (Figure 6), suggesting this was indeed the case.

### CE-binding antibodies induced by synthetic E5 peptide

**Figure 7:**
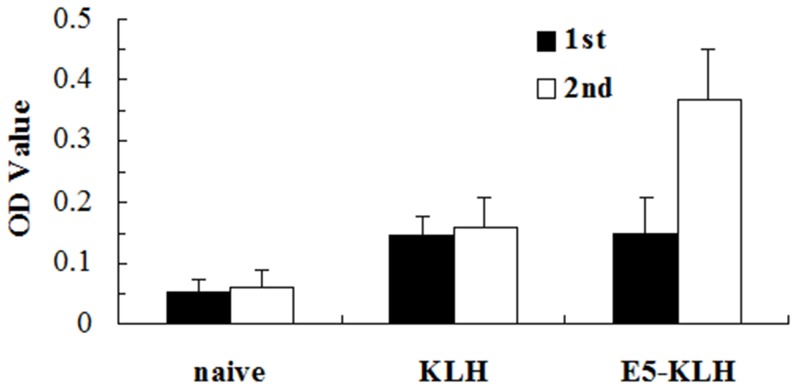
Anti-CE antibodies titres in mice immunized with E5 synthetic peptide. CE-binding IgG in the sera of mice (n = 5) immunized twice (50 µg per mouse) with KLH conjugated E5 peptide or with KLH as a control and naïve mice were detected by ELISA as described in Methods. IgG levels are shown prior to and after each immunization (1^st^ filled bars and 2^nd^ open bars). The OD value of each sample in triplicate was read 15 min after addition of substrate and the mean±SD was determined. Significance was determined by a two-tailed, Mann-Whitney test. (P = 0.0079).

To further evaluate whether E5 phage displayed peptide was a mimotope of the CE protein and could induce CE-binding antibodies, mice were immunized with either KLH-conjugated E5 peptide or unconjugated KLH as a control. E5 and CE-binding antibodies were detected after first and second immunization. Significantly more CE-binding IgG was detected in sera from mice immunized with the KLH-conjugated E5 peptide compared with KLH-immunized or unimmunized mice (Figure 7, Mann-Whitney U = 0, P = 0.0079).

### Reactivity of E5 peptide to TB patient sera

**Figure 8:**
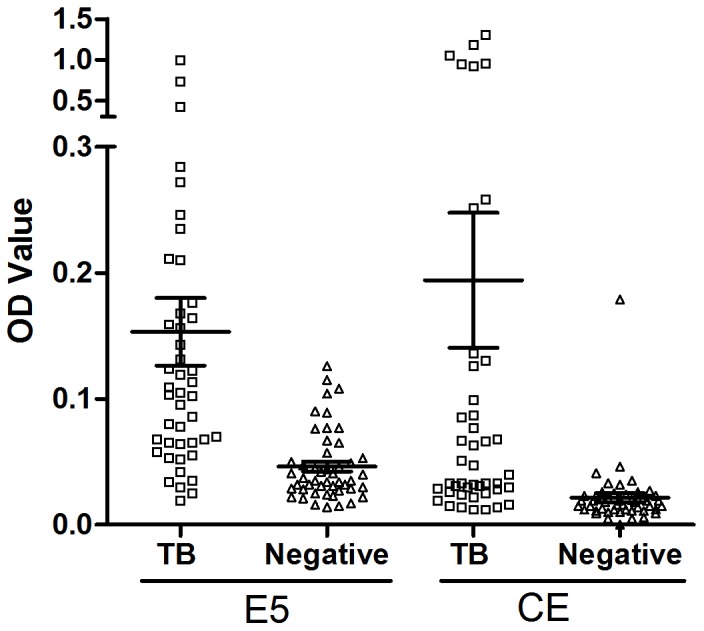
Scatter dot plot representation of the distribution of IgG levels to the peptide E5 and CE protein in TB patients (□) and negative control (▵). (Error bars: 95% confidence interval for mean). The plates were coated with 4 µg peptides E5 ml^−1^ or 1 µg CE protein ml^−1^ per well, as described in Methods. Sera from TB patients (n = 44) and healthy individuals (n = 48) were tested. The results are shown as the mean OD value of each triplicate sample read 20 min after the addition of substrate. Significance was determined against the negative controls group of E5 peptides and CE protein (t = 4.086, P<0.0001; t = 3.359, P = 0.0011). The sensitivity and specificity of E5 peptide and CE protein were 54.5% and 91.7%, 34.1% and 97.9%, respectively, with a cut-off value at mean +2SD of the healthy controls.

The reactivity of the E5 peptide against TB patient sera was evaluated by indirect ELISA. Reactivity was compared with that of the CE protein. Significantly higher titres of E5 peptide-binding antibodies were found in the sera of TB patients compared with those of healthy individuals (t = 4.086, P<0.0001). Similar results were found for CE protein (t = 3.359, P = 0.0011) (Figure 8). Using a cut-off value at mean +2SD of the healthy controls values, the sensitivity and specificity of E5 peptide were 54.5% and 91.7%, and for the CE protein were 34.1% and 97.9%, respectively. The area under the receiver operating characteristic (ROC) curve (AUC) of E5 peptide and CE protein were evaluated using the panel of TB and negative controls sera. AUC were determined to be 0.841 and 0.845, for E5 peptide and CE protein respectively, and according to the pair-wise comparison, there was no significant difference between the AUC of E5 and CE (Z = 0.0723, P = 0.942). Thus, the diagnostic capability of the E5 peptide was comparable to that of the CE protein.

**Figure 9:**
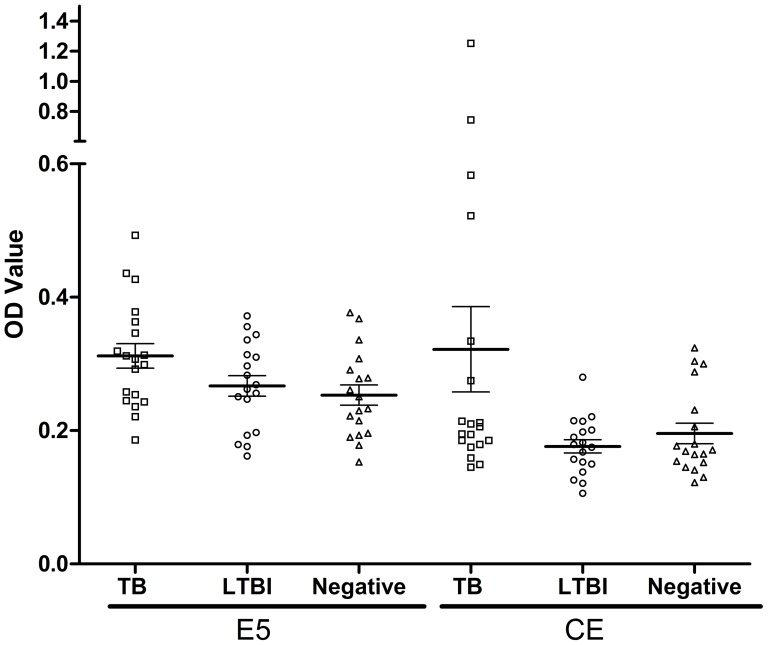
Scatter dot plot representation of the distribution of IgG levels to the peptide E5 and CE protein in TB patients (□), LTBIs (○) and negative controls (▵). (Error bars: 95% confidence interval for mean). The plates were coated with 4 µg peptides E5 ml^−1^ or 1 µg CE protein ml^−1^ per well, as described in Methods. Sera from TB patients (n = 19), LTBIs (n = 18) and healthy individuals (n = 18) were tested. The results are shown as the mean OD value of each triplicate sample read 20 min after the addition of substrate. Significant differences for three groups were determined of E5 peptides and CE protein (F = 3.522, P = 0.0368; F = 4.026, P = 0.0237).

To extend the data, we also assessed if the antibody against the E5 peptide could detect differences between active and latent tuberculosis. Blood samples from 19 active TB patients, 18 LTBIs and 18 healthy individuals, were tested using E5 peptide and CE coated plates. The mean values of the TB, LTBI and control groups were found to be significantly different for both E5 and CE upon one-way ANOVA analysis (F = 3.522, P = 0.0368; F = 4.026, P = 0.0237) (Figure 9). Using Tukey's multiple comparison tests, significant difference was found between active TB vs. healthy controls for E5 peptide like before (P<0.05), but not between different TB stages (active TB vs. LTBI) and LTBI vs. control; for CE, however, the significance was only found between active TB vs. LTBIs (P<0.05), not between any TB disease stage (active TB or LTBIs) vs. control, suggesting that the CE protein can discriminate active TB from LTBI, and the responses to LTBIs of E5 peptide and CE are different.

## Discussion

Uses of the CFP10 and ESAT-6 proteins for the detection of TB infection are well established [30], [31]. Despite this, and although vigorous antibody responses have been observed for CFP10 and ESAT-6 protein [32], serodiagnosis of TB using CFP10 and ESAT-6, either separately or combined, remain unsatisfactory[33], [34]. This may be influenced by the antigenic variability or the cross reaction with other antigens [35]. Using peptides representing the pertinent B-cell epitopes as a replacement of whole recombinant proteins has many benefits in the diagnostic tests [36]. Synthetic peptides can be produced cheaply, are well-defined and highly reproducible, and could potentially avoid cross reactivity [37], [38]. One of the B-cell epitope of ESAT-6 has been confirmed at position 3 to 15 before [15], but to date, other B-cell epitopes of ESAT-6, especially of the CE protein, have not been tested directly.

To identify B-cell epitopes representing the CE protein of MTB, we generated pAbs directed against CE and used these as a target for biopanning with a linear 7 peptide phage display library [39], [40]. After four rounds of biopanning, phage clones were randomly selected, identified and nine different sequences were detected (see Table 1). Moreover, 6 of the 18 phage clones were identified carrying heptamer-peptides belong to a consensus sequence (FTHEAFG, named E1). This may be the result of higher specificity or binding affinity to the target of the peptide [41]. However, according to the result of sequence alignment, no recurring sequence and no more than two amino acids analogue of E1 to CE were found (see figure 1). The relative binding efficiency of E1 tested by phage-ELISA was also not markedly higher than other clones, indicating that E1 may be an ineffective mimotope of CE protein and this frequency may have arisen as a result of nonspecific biopanning.

Harboe *et al.* [15] have confirmed that antibodies formed by immunizing with a 23-mer peptide representing positions 40 to 62 of ESAT-6 could react with the native ESAT-6, indicating that a B-cell epitope may exist in this region. Nahtman *et al.* [17] also confirmed that the Q54OD8 peptides (position 53 to 66) of ESAT-6 were recognized by TB patients. The precise B-cell epitope in this region, however, was not defined. According to our sequence alignment results, a recurring sequence WDAT was found for 5 phage clones E5, E6, E7, E9, and E18, which was consistent with the 163^rd^–166^th^ amino acid sequence of CE (see Figure 1) and exactly matched the 58^th^–61^st^ sequence of ESAT-6 protein. According to the structure modeling figure of CE, the amino acid sequence WDAT should be located on the surface of the space structure of CE (Figure 2). Furthermore, the recognition of E5, E6, E18 phage-displayed peptides by the anti-CE pAbs (see figure 3) and the competitive ELISA of CE protein and E5, E6, E18 phages (see figure 4) all indicated that the WDAT is likely the core of a linear B-cell epitope of CE. The 1^st^–5^th^ amino acid KWDAT of E5 phage displayed peptide was consistent with the 57^th^–61^st^ sequence of ESAT-6, and the binding affinity of E5 was the highest of all phage clones tested (see figure 3), suggesting that the Lysine residue may be involved in the composition of the B-cell epitope. The competitive inhibition results generated using synthesized biotin-labeled peptides (E5BIO, E6BIO and E18BIO) with CE and ESAT-6 proteins (see figure 6) provide further evidence that both the CE protein and the ESAT-6 protein alone can specifically inhibit the binding of the identified mimotope to anti-CE pAbs. It has been reported that mimetics with higher affinities could make mimotopes with better immunological activities [42]. Significant CE-binding IgG was also detected in mice immunized with KLH-conjugated E5 peptide, and we may therefore conclude that KWDAT should be a linear B-cell epitope of the CE protein.

From the sequence alignment, no analogue of the E5, E6, and E18 phage clones to CFP10 protein was found, but according to the competitive ELISA results, CFP10 protein can effectively inhibit the peptides binding to anti-CE pAbs. Since there are presumably CFP10-specific antibodies within the anti-CE pAbs, these peptides may react with the anti-CFP10 antibodies binding to the conformational epitopes of CFP10 protein, meaning that these peptides may represent a conformational mimotope of CFP10. This hypothesis requires further exploration.

To evaluate if TB patients sera that react with CE protein can also bind E5 peptide, and if these can be used to the serodiagnosis of TB, E5 peptide and CE protein were coated on the 96-well ELISA plates and samples from TB patients and healthy individuals were assayed [43], [44]. PPD tests of healthy people vaccinated with BCG may be positive, but because CE is deleted in all BCG vaccine strains, CE antibodies should not be present [32]. To evaluate if E5 responses of TB patients were comparable to those against the CE protein, and if these can differentiate TB patients from healthy BCG-vaccinated individuals, we compared responses within sera from active pulmonary TB patients and healthy individuals, all of whom were BCG-vaccinated and were also PPD-positive. Similar results were obtained with CE protein and E5 peptide, indicating the possibility of using the E5 peptide in the serological diagnosis of TB. However, latent TB infections (LTBIs) were not excluded from the healthy individuals in our primary analysis. To extend our data, additional blood samples from recruits divided into three groups according to the responses in their T-SPOT TB test results were evaluated against the E5 peptide and CE protein. These analyses suggest that the anti-E5 antibodies could be a biomarker of TB infection, detecting differences between TB patients and non-TB infections, and the CE protein could be a marker of active disease, discriminating TB patients from LTBIs. Furthermore, of the serum samples selected, immunocompromised patients and/or those infected by environmental *Mycobacterium marinum*, *Mycobacterium kansasii*, *Mycobacterium szulgai*, as well as *Mycobacterium bovis* infection, all of which also have CE protein, were not included. Thus, analyses of additional samples are required to confirm the conclusion and find out if E5 can therefore be used for the TB diagnosis.

In conclusion, B-cell linear epitopes of CE protein predicted using phage display were confirmed by sequence alignment, binding affinity and specificity detection, generation of polyclonal mouse sera and finally, by specific detection of TB patient sera. Together, our data indicate a promising candidate for the serodiagnosis of TB, warranting further studies of the E5 peptide.
